# A Predictive Model Based on Pyroptosis-Related Gene Features Can Effectively Predict Clear Cell Renal Cell Carcinoma Prognosis and May Be an Underlying Target for Immunotherapy

**DOI:** 10.1155/2022/6402599

**Published:** 2022-07-08

**Authors:** Yufu Wang, Jinhui Liu, Lishuo Zhang, Yifei Li

**Affiliations:** Department I of Urology, The Second Affiliated Hospital of Harbin Medical University, No. 246 Xue Fu Road, Nangang District, Harbin 150001, China

## Abstract

**Methods:**

The clinical information and RNA-seq data of ccRCC patients were collected from the TCGA dataset to first explore differential pyroptosis-related genes (PRGs). Univariate Cox regression and consensus clustering were applied to identify ccRCC subtypes. The prognostic PRGs were subjected to LASSO regression analysis to establish a prognostic model and to investigate its value and function. Finally, the relationship of the model immunity checkpoints and immunity infiltration was assessed.

**Results:**

The receiver operating characteristic (ROC) showed that the 1-year, 3-year, and 5-year prediction rates of the prognostic model were 0.715, 0.693, and 0.732, respectively. The high-risk group had lower overall survival and higher stage than the low-risk group. Functional enrichment analysis showed that PRGs were significantly enriched mainly in the PPAR pathway, inflammatory pathway, and immune activity. ccRCC patient prognosis correlates with immune components in the microenvironment, and immune checkpoint molecules are significantly expressed in the high-risk group. Immunotherapy may be effective in the high-risk group.

**Conclusion:**

Pyroptosis-related gene has an important impact on the progression of ccRCC and can be used as an independent predictor of patient prognosis. In addition, immune checkpoint molecules are significantly upregulated in high-risk populations, which may be a potential target for immunotherapy.

## 1. Introduction

ccRCC is the most familiar histologic type of renal cell carcinoma (RCC), accounting for about 85% of whole main tumors of kidney. Although surgical treatment can achieve better therapeutic results in early phases, this cannot be always monitored until the later stages of the disease. In addition, because the disease usually progresses rapidly and 30–40% of patients with localized disease still experience recurrence and metastasis after surgical resection, the prognosis for patients is generally poor [1, 2]. The neoplasm immunity microcircumstances exert a crucial part in progression of many tumors, as well as usage in targeted therapy against the tumor immune microenvironment is considered to be a promising therapeutic modality [3]. However, as the neoplasm immunity microcircumstances can be a complicated ecosystem, the current characterization of the composition and cellular status of infiltrating ccRCC immune cells has not been fully elucidated [4]. Therefore, understanding immune cell infiltration in the ccRCC microenvironment from a pyroptosis perspective is significant for developing targets for potential immunotherapy.

Inflammation is the initiation of certain types of malignant tumor changes, which exerts a vital part in development of many tumors [5]. Inflammation is conductive to malignant cellula proliferating and is an important player in tumor escape and progression [6]. Pyroptosis is a programmed death induced by various stimuli that trigger the inflammasome [7]. Pyroptosis activation leads to cytoplasmic swell, plasmalemma dissolution, and chromosome dispersion and promotes the discharge of cellular substances (IL-1*β* as well as IL-18) [8]. There are two main pathways for pyroptosis to trigger inflammation: the canonical inflammasome pathway and noncanonical inflammatory corpuscle path. Canonical inflammatory corpuscle path triggers pyroptosis via caspase-1-mediated secretion of proinflammatory factors (pro-IL-18 as well as pro-IL-1*β*) leading to cell lysis. The noncanonical inflammatory corpuscle path induced pyroptosis mainly via activating caspase-11 as well as caspase-4/5 to cleave gasdermin D (GSDMD) [9, 10].

In recent years, pyroptosis is shown to be related to many human illness, but its relationship with cancer prognosis has been elusive. The main reason is that pyroptosis has a double part among cancer progression [11–14]. In one aspect, pyroptosis triggered by inflammasomes leads to the release of a large number of cytokines that alter the immune microenvironment and help tumors evade immune surveillance [15, 16]. For example, Barber et al. found strong caspase-1 expression during Barrett's esophagus (BE) to esophageal adenocarcinoma (EAC), confirming that stromal caspase-1 expression is closely related to the development of esophageal adenocarcinoma [17]. Marta data showed GSDMB expression could be upregulated in breast cancer patients contrasted to normal breast tissue, high levels of which expression correlated with decreased survival and increased metastasis [18]. On the other hand, cytokines produced by pyroptosis-related gene also activated immunity system to improve efficiency of cancer immunotherapy. For example, under hypoxia, PD-L1 uses caspase-8 to specifically cleave GSDMC and produce the N-terminal domain, triggering pyroptosis and promoting tumor necrosis [19]. Cui et al. showed that MST1 may inhibit the progression of glandular ductal adenocarcinoma (PDAC) cell proliferation and migration and invasion through reactive oxygen species- (ROS-) induced pyroptosis [20]. Thus, it can be vital to study role and mechanism of pyroptosis in cancer development for treatment as well as prognosis.

Currently, there are increasing discoveries about the mechanism and function of pyroptosis in tumors. However, the function of pyroptosis in ccRCC is not clear. We established a pyroptosis-related prognosis model according to the resulting pyroptosis-related genes (PRGs) to understand the potential value in ccRCC, which offers novel thoughts for intervening in the diagnostics and therapy among patients with ccRCC.

## 2. Materials and Methods

### 2.1. Data Source

**Figure 1:**
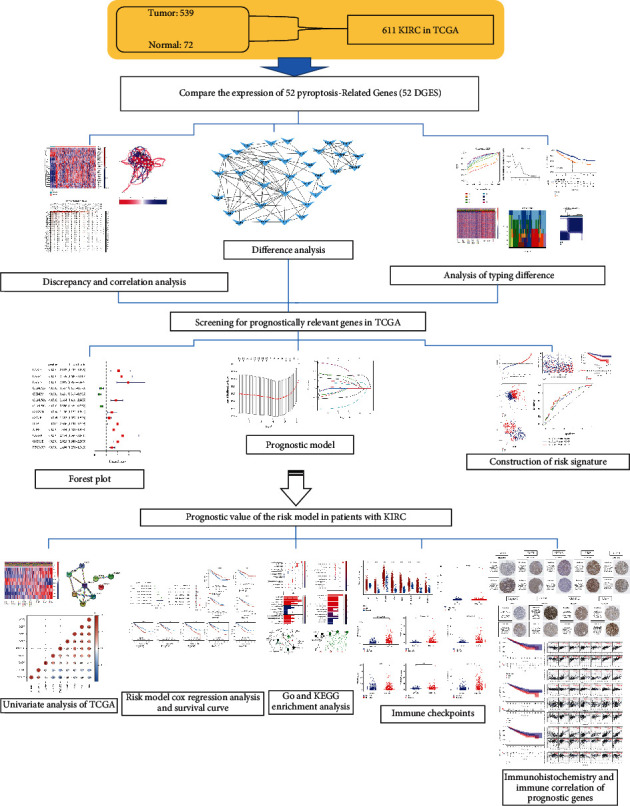
Analysis flowchart for the research.

The research devise and workflow are indicated in Figure 1. As of December 31, 2021, the cancer genome profile (TCGA) databases (https://http://portal.gdc.cancer.gov/repository) RNA sequencing (RNA-seq) data and corresponding clinical features were obtained from 539 ccRCC patients and 72 normal renal tissue. Expression information of the two datasets was standardized to values of millions of fragments per kilobase (FPKM) prior to contrast.

### 2.2. Identification of Discriminatively Expressed Relevant Genes

According to differentially expressed pyroptosis-related gene relevant genes, ccRCC patients were classified into different groups using consensus clustering. A consensus cluster consisting of differentially expressed PRGs was constructed using the “ConsensusClusterPlus” package, and we performed 1,000 replicates to ensure the stability of our classification. Kaplan-Meier study was conducted with R “survival” package to assess differences in prognosis of ccRCC patients in distinct parts. Typing heat visualizes this prognostically relevant gene expression difference and analyses this relationship based on clinical-pathological parameters.

### 2.3. Consensus Clustering of Differentially Expressed PRGs and Prognostic Relevance in ccRCC

Based on discriminatively expressed PRGs, ccRCC sick persons could be classified into different groups using consensus clustering. A consensus cluster consisting of differentially expressed PRGs was constructed using the “ConsensusClusterPlus” package, and we performed 1,000 replicates to ensure the stability of our classification. Kaplan-Meier study was conducted with R “survival” package to assess differences in prognosis among ccRCC patients in distinct parts. The typing heat map visualizes this prognostically relevant gene expression difference and analyses this relationship based on clinical-pathological parameters.

### 2.4. Construction of Prognostic Model of ccRCC Based on PRGs

To evaluate the process value of genes associated with pyroptosis-related gene, we next used a Cox regression study to determine prognostic value of PRGs and identified genes which were remarkably related to overall survival of ccRCC sick persons. Then, the least absolute shrinkage and selection operator (LASSO) regression algorithm was used for character selection, and ten times cross-validation was adopted. The R software package “glmnet” was used for aforementioned study. After concentration and normalization of the TCGA expression data (applying the “proportion” function in R), a risk score = coef (PRG1) expr (PRG1) + coe2f (PRG2) expr (PRG2) + ⋯+coef (PRG9) expr (PRG9) was calculated. Where coef denotes the coefficient, coef (PRGn) is the coefficient of PRGs associated with prognosis, and expr (PRGn) is the expression of PRGs. The risking value for every sick persons was counted using this formula; and then, TCGA ccRCC sick persons were separated in low and high-risking parts based on the middle risking value. In the end, ccRCC surviving distinctions among two parts could be compared via Kaplan-Meier analysis. PCA and t-SNE analyses of prognosis-related gene models could be conducted with “Rtsne” and “ggplot2” R packages. ROC curve analysis was performed at 1, 3, and 5 years using the “survival,” “survminer,” and “time-ROC” R software packages.

### 2.5. Cox Regression Analysis of Prognostic Models and Correlation with Clinical Pathological Characteristics

In order to further elucidate underlying function of PRGs among ccRCC, we used pheatmap to construct nine prognostically relevant gene risk heat maps. Prognostic-associated gene PPI networks were constructed using STRING and subjected to Spearman correlation analysis. The prognostic model was subjected to Cox regression analysis, and forest plots were drawn through “forestplot” R package. In addition, R “suivival” package could be applied for prognostic gene KM survival curve distribution.

### 2.6. Gene Set Enrichment Analysis

CcRCC patients in the TCGA queue were separated in two parts according to middle risking value. Screening among low and high risking parts was based on definite standards (|log2FC| ≥ 1 as well as FDR *q* value < 0.05). The “clusterProfiler” R package was used for GO and KEGG study of genes associated with prognosis. In addition, KEGG pathway enrichment was performed using the Cytoscape plugin “ClueGO.”

### 2.7. Immunohistochemical Staining Characteristics of Prognostic Genes

Using the HPA (the mankind protein atlas) database (https://www.proteinatlas.org), immune-histochemical staining images of prognostic genes among ccRCC tumor tissues as well as normal tissues were studied. HPA database is use of the integration of various genomics technologies to draw a map of all human proteins in cellulas, tissues, and organs, including antibody-based image formation, MS-based proteomics, transcription omics, and systematic biology, all of which have free open access to data.

### 2.8. Correlation between Prognosis Genes as well as Immunity Infiltration

We used R language ESTIMATE algorithm using the Rx64 version 4.0.5 feature to count proportion of immunity and stromal cellulas in the TME for every specimen. Stromal core was positively related to matrix cells, immunity core value was positively related to immune cells, and the sum of the two was regarded as the total score. If the corresponding score in the microenvironment is higher, the corresponding content is higher. We next studied association among prognosis genes as well as immunity infiltration by TIMER. TIMER is a dependable database for analyzing the abundant tumor-infiltration immunity cellulas.

### 2.9. Experimental

Wilcoxon test was applied to contrast 2 parts, as well as Kruskal-Wallis measurement was applied to contrast more than 2 parts. Spearman was used for relevance analysis between genes and gene expression. Kaplan-Meier analyses were applied to assess overall survival, as well as log-rank tests were applied to contrast overall survival parts. The Mann–Whitney test was used when comparing immune cell infiltration and immune pathway activation between the two groups. Mann–Whitney measurement was applied to compare the infiltration of immune cells as well as activation of immunity pathways among 2 parts. Gene expression data and all statistical analysis could be completed in R (v4.1.1).

## 3. Results

### 3.1. The Study Procedure of the Research Is Indicated in Figure 1

We summarize this work into the following flowchart.

### 3.2. Identification of PRGs among Normal as well as Cancer Tissue

**Figure 2:**
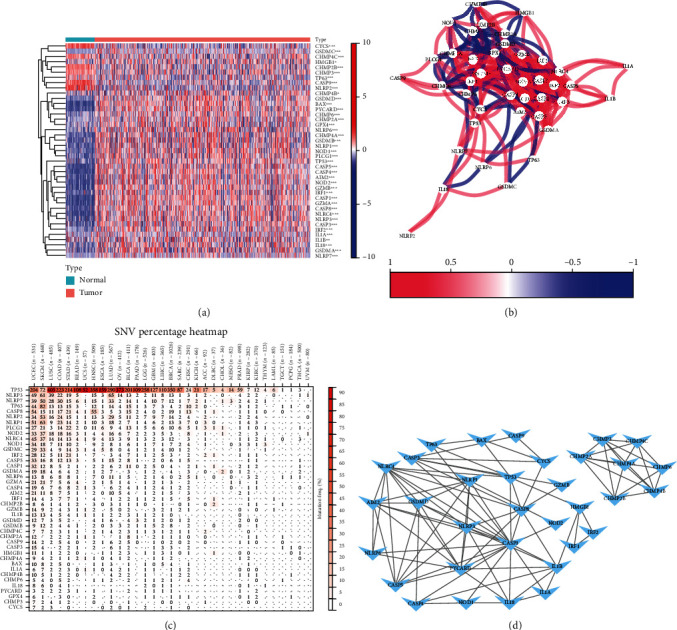
Discriminatively expressed PRGs and their mutual effects. (a) Heat map of discriminative expression of pyroptosis-related gene among normal as well as cancer tissues. ∗*P* < 0.05; ∗∗*P* < 0.01; ∗∗∗*P* < 0.001. (b) The PPI net showed mutual effects with PRGs (mutual effect scores = 0.7). (c) Relevant networks of PRGs. (d) Heat map of pyroptosis differential gene single nucleotide variation (SNV) expression in the pan-cancer spectrum.

Contrast expression standards of 52 PRGs among 72 normal tissues as well as 539 tumor tissues from TCGA-ccRCC data and discovered that all 41 cell PRGs were discriminatively expressed among cancer as well as neighboring noncancer tissues (*P* < 0.01). Nine of these genes (NLRP2, TP63, CYCS, CASP9, IL1A, CHMP2B, CHMP4C, CHMP3, and IL1B) were downregulated, while 32 genes (HMGB1, CHMP4B, IRF2, CHMP2A, CHMP6, TP53, GPX4, CASP3, PLCG1, NOD1, GSDMD, CASP8, IL18, CHMP4A, IRF1, BAX, NLRP1, CASP4, NLRP3, GSDMA, NLRP6, CASP1, NLRC4, GSDMB, PYCARD, GSDMC, NLRP7, NOD2, GZMB, CASP5, AIM2, and GZMA) were upregulated in cancer tissues. Discriminative gene expression in 2 parts is showed as a heat map (Figure 2(a), blue: low expression standard; red: high expression standard).

To further explore mutual effects of the PRGs, we performed protein-protein interactions (PPIs). The results showed that NLRP1, GSDMD, NLRC4, CASP1, CASP3, NLRP3, CASP8, PYCARD, CHMP4A, and AIM2 may be central genes (Figure 2(b)). In addition, the correlation net of overall pyroptosis-related gene differentially associated genes can be indicated in Figure 2(c) (red: positive correlation; blue: negative correlation). Finally, pan-cancer expression profiling revealed the presence of single nucleotide variants (SNVs) in the majority of clear cellular cancer of kidney (Figure 2(d)).

### 3.3. Consensus Clustering Identified Two ccRCC Clusters

**Figure 3:**
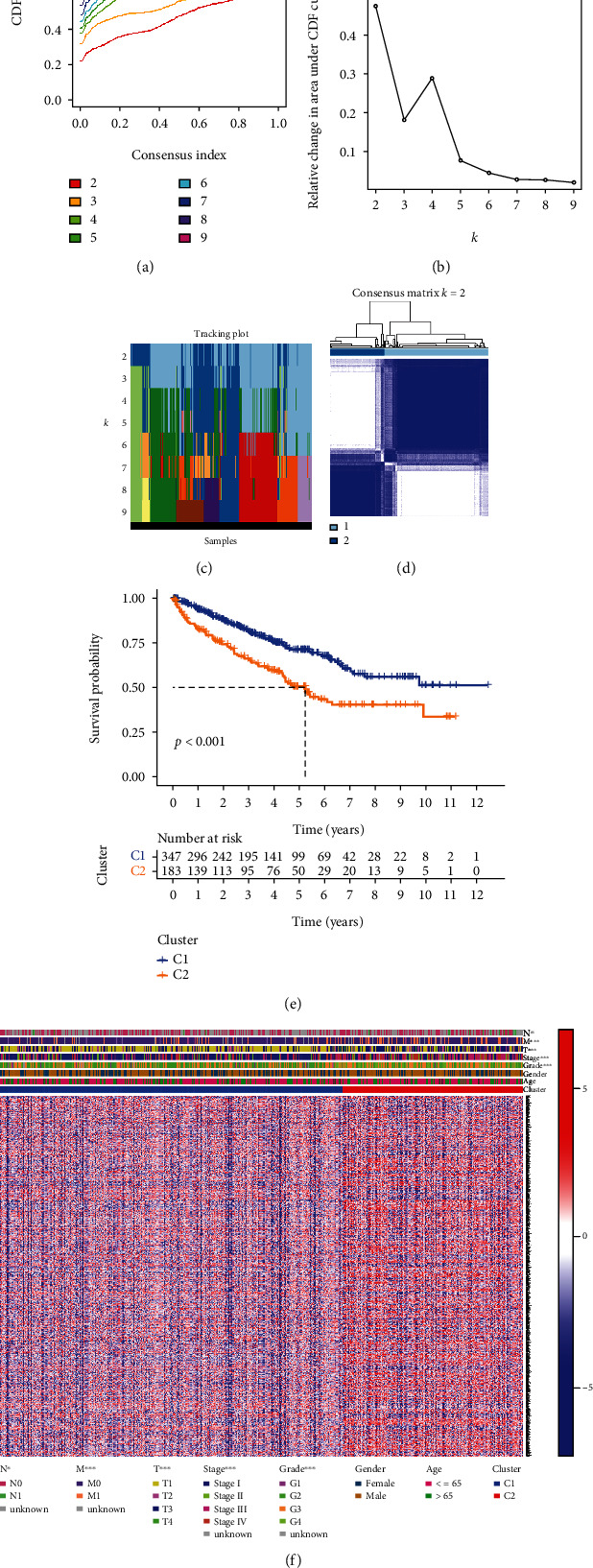
TCGA cohort subtype classification and relationship with different clinical characteristics and survival. (a) Agreement cluster cumulative distribution function (CDF) with *k* = 2 ~ 9. (b) Relative alert in the area below CDF profile for *k* = 2 to 9. (c) Distribution of each sample for *k* between 2 and 9. (d) When *k* = 2, the ccRCC cohort from TCGA is split into two distinct clusters. (e) Kaplan-Meier overall survival profile of two. (f) Heat map showing distribution of clinicopathological variables between the two clusters.

To find out link among PRGs as well as different groupings of ccRCC, we conducted an agreement clustering study on 530 ccRCC sick persons with complete clinical data in TCGA queue. By adding cluster variable (*k*) from 2 to 10, we discovered *k* = 2 was the most appropriate selection to partition the ccRCC sick person queue in 2 clusters (Figures 3(a)–3(d)). Kaplan-Meier curves showed that cluster two had a significantly worse overall survival time than cluster one prognosis, which was statistically significant (*P* < 0.001, Figure 3(e)). In addition, the gene expression curves and clinic characters are indicated with a heat map (abscissa represents the sample, and ordinate represents the typing differential gene). Characteristics included gender, age (≤65 or >65 years), stage (I-IV), grade (G1-G4), TNM stage, and cluster subtype. Compared with C1, we found that C2 could be remarkably related to a higher stage, grade, and TNM stage (Figure 3(f)).

### 3.4. Construction and Validation of Nine Pyroptosis-Related Gene Models

**Figure 4:**
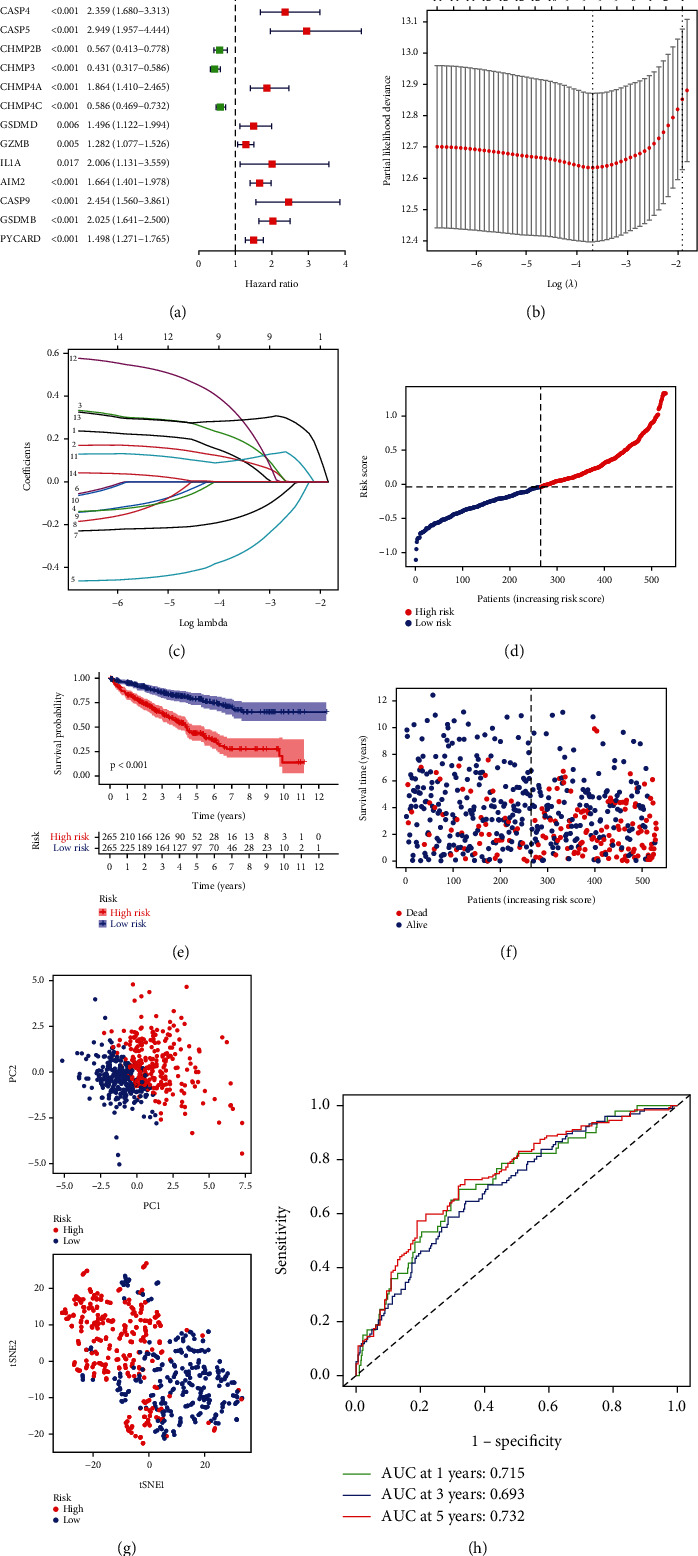
Establishment of risking model in TCGA cohort. (a) Cox univariate study to determine the forest plot of the association between 14 PRGs and OS. (b) The best nine genes associated with OS were screened in LASSO regression model. (c) LASSO coefficient distribution determined by optimal lambda. (d) Distribution of patients according to risking value. (e) Kaplan–Meier profiles for overall survival in high as well as low risk patients. (f) Surviving condition of every sick person (low risk population: left dashed line; high risk population: right dashed line). (g) PCA and t-SNE plots of ccRCC according to risking score. (h) ROC profile demonstrates the prediction efficiency of risking value.

All 530 corresponding sick persons are with entire surviving information in ccRCC sample. Univariate Cox regression study was applied as an initial screening for survival relation genes. Fourteen genes (CASP3, CASP4, CASP5, CHMP3, CHMP4A, CHMP2B, CHMP4C, GSDMD, GZMB, IL1A, AIM2, CASP9, GSDMB, and PYCARD) that satisfied standards of *P* < 0.05 were kept for next study, of which 11 genes (CASP3, CASP4, CASP5, CHMP4A, GSDMD, GZMB, IL1A, AIM2, CASP9, GSDMB, and PYCARD) increased the risk of disease, while the other three genes (CHMP3, CHMP2B, and CHMP4C) were protective genes (Figure 4(a)). The prognostic model constructed by nine genes could be selected according to best score by LASSO Cox regression analysis (Figures 4(b) and 4(c)). Risking value can be calculated below: Risking score = (0.121CASP3 exp.) + (0.107CASP4exp.) + (0.207CASP5exp.) + (0.098AIM2exp.) + (0.331CASP9exp.) + (0.286GSDMBexp.) + (0.0003PYCARDexp.) (−0.350CHMP3exp.) + (−0.172CHMP4Cexp.). They were separated in high risk as well as low risk parts based on middle risking value (Figure 4(d)). PCA study as well as t-SNE analysis indicated sick persons with distinct risking could be well distinguished in 2 parts (Figure 4(g)). Kaplan-Meier curves show remarkably worse OS in high-risk part than in the low-score part (*P* < 0.001, Figure 4(e)). It was indicated by Figure 4(f) that sick persons in high-risk value part could be strongly related to a high risking of death rate, but sick persons in low risk part had surviving possibility. Moreover, ROC profile study was conducted to evaluate discrimination ability of gene features using R package “survival ROC” (Figure 4(h)).

### 3.5. Correlation between Prognosis Models as well as Clinical Pathological Factors

**Figure 5:**
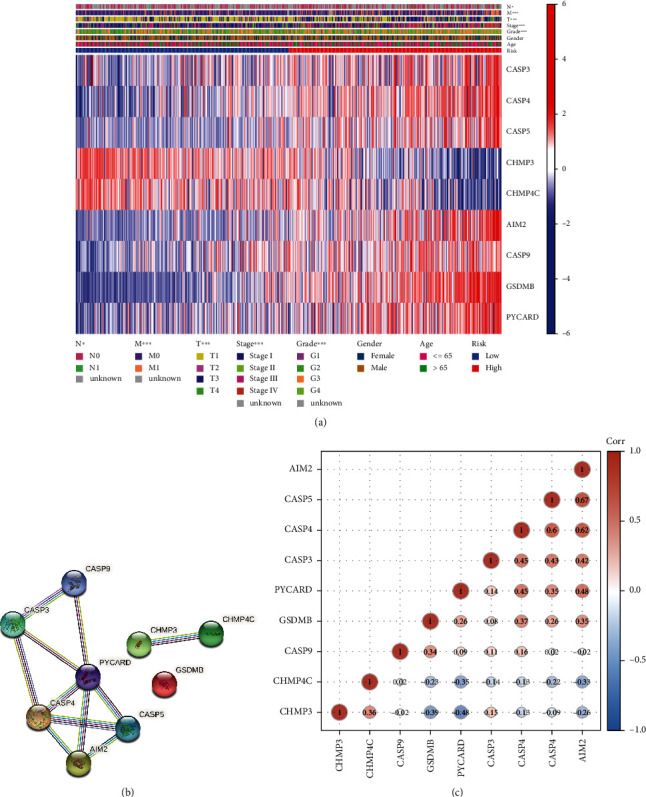
Screening of prognostic risk genes. (a) Heat map was used for link among clinical pathological character as well as risking parts (green: low expression; red: high expression, *P* < 0.05). (b) PPI net shows interaction of risk genes (lines represent interacting genes). (c) Spearman correlation analysis among gene expressions.

We studied the association of PRG expression profiles using different risk groups as well as clinical pathological features using heat maps. The results showed that a significant difference in tumor phase was examined among low-risk as well as high-risk patients, like more stage IV as well as fewer T1 specimens in high risk sick persons (Figure 5(a)), *P* < 0.05. Nine gene interactions in the prognostic model were explored using PPI and Spearman correlation analyses (Figures 5(b) and 5(c)).

### 3.6. Prognostic Models Are Independent Prognosis Elements

**Figure 6:**
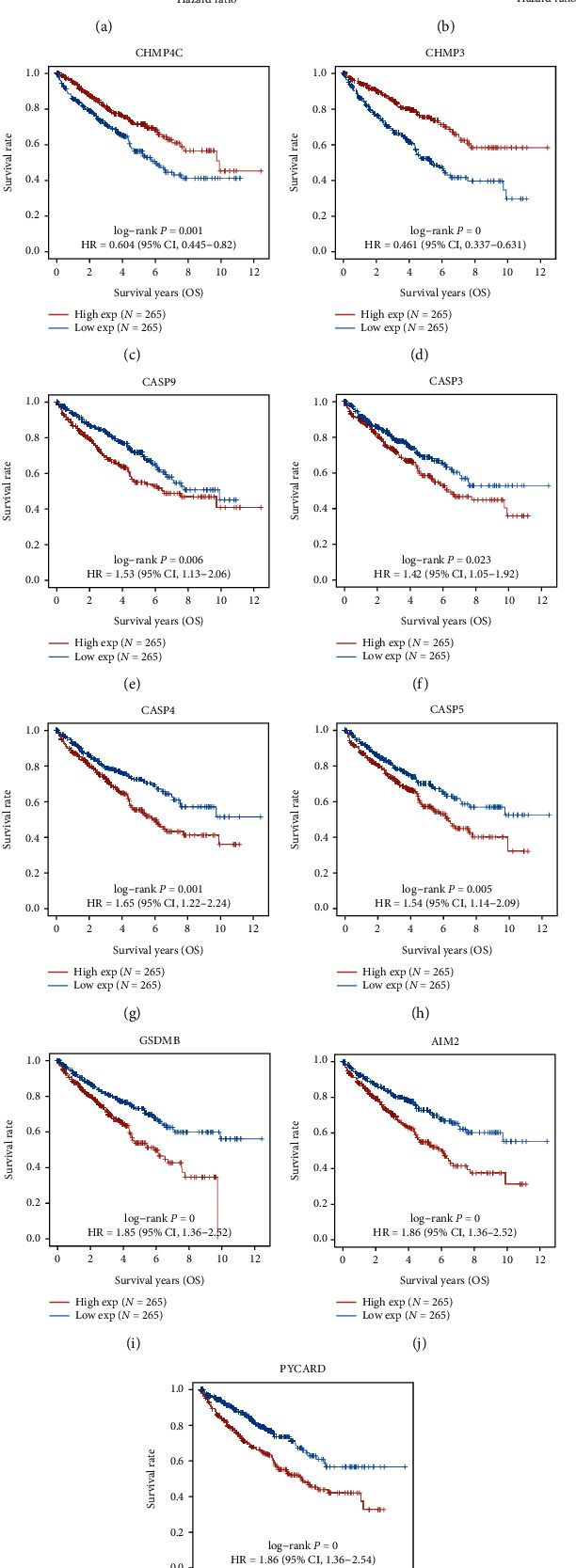
Procedure value of prognosis models. (a, b) Univariate as well as multivariate Cox regression analysis of risking scores. (c) Kaplan-Meier surviving profile for CHMP3. (d) Kaplan-Meier surviving profile for CHMP4C. (e) Kaplan-Meier surviving profile for CASP9. (f) Kaplan-Meier surviving curve for CASP3. (g) Kaplan-Meier surviving curve for CASP4. (h) Kaplan-Meier surviving curve for CASP5. (i) Kaplan-Meier surviving curve for GSDMB. (j) Kaplan-Meier surviving curve for AIM2. (k) Kaplan-Meier survival curve for PYCARD.

We used Cox regression analysis to evaluate whether prognosis model could act as an independent prognosis element. Univariate Cox regression study showed that age, grade, TNM, and risk score were related to prognosis, *P* < 0.001 (Figure 6(a)). Second, a multivariate Cox regression study indicated risk value, age, and M phase were independent elements influencing the prognosis among ccRCC sick persons, *P* < 0.001 (Figure 6(b)). In addition, Kaplan-Meier survival curves for gene expression in the prognostic model are indicated in Figures 6(c)–6(k). The consequences indicated there were seven prognostic genes with high expression negatively relevant to surviving rate of ccRCC sick persons (CASP3, CASP4, CASP5, AIM2, CASP9, GSDMB, and PYCARD), and two prognostic genes with high expression positively correlated with surviving rate of ccRCC sick persons (CHMP3 and CHMP4C), *P* < 0.05.

### 3.7. Enrichment Functional Analysis of Prognostic Models

**Figure 7:**
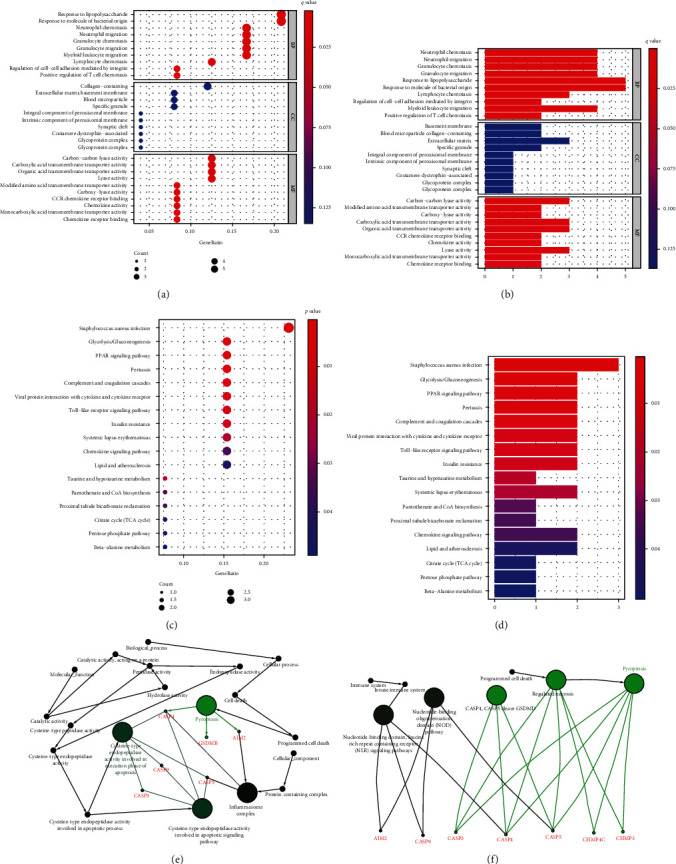
Function study according to PRGs among 2 risking parts in TCGA queue. (a, b) GO enrichment analysis of PRGs. (c, d) KEGG enrichment study of PRGs. (e) Consequences of ClueGO GO: activation of the inflammatory response. (f) Consequence of ClueGO KEGG path enrichment: triggering pyroptosis.

In order to further find out distinctions in gene function as well as paths among risk genes, KEGG and GO function enrichment studies of the risk genes could be performed. GO results indicated that PRGs were primarily referred to inflammatory responses, immune activity, chemokine-mediated signaling pathways, and inflammatory cell chemotaxis (Figures 7(a) and 7(b)). Moreover, KEGG path analysis showed PRGs could be highly abundant among PPAR signaling pathway, Staphylococcus aureus infection, glycolysis/gluconeogenesis, and Toll-like receptor signaling pathway (*P* < 0.05; Figures 7(c) and 7(d)). ClueGO showed a network diagram of the correlation between prognostic genes and pyroptosis (Figures 7(e) and 7(f)).

### 3.8. Prognostic Risk Scores Correlate with the Expression of Immunity Checking Points

**Figure 8:**
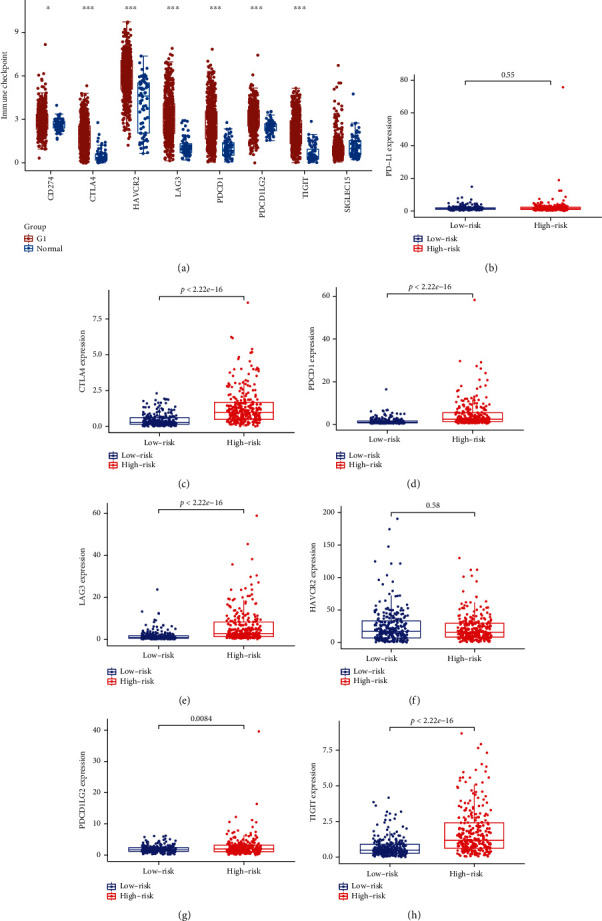
Prognostic risk genes are associated with immune checkpoints. (a) Expression of immune checkpoints in renal clear cell carcinoma (G1) and normal tissues. (b–h) Contrast to low risking part, expression standards of PD-L1 (CD274), CTLA4, PDCD1LG2 (PD-L2), LAG3 (CD223), TIGIT, HAVCR2 (TIM-3), and PDCD1 (PD-1) could be remarkably upregulated in high risk part, *P* < 0.001.

In order to determine if our risking model can reflect the status of the immune microenvironment and provide guidance for immunotherapy response, we extracted 539 renal clear cell carcinoma tissues as well as 59 normal specimens through TCGA database. Results of eight immune checkpoint tests are shown in Figure 8(a), and G1 represents renal clear cell carcinoma samples. We then analyzed the differential expression of immunity checking points among sick persons at different risks. In high risk group, PDCD1 (PD-1), CTLA4, PDCD1LG2 (PD-L2), TIGIT, and LAG3 (CD223) could be significantly highly expressed, *P* < 0.05 (Figures 8(c)–8(h)). However, PD-L1 (CD274) and HAVCR2 (TIM-3) could not be significantly distinct, with *P* scores of 0.55 as well as 0.58, separately (Figures 8(b) and 8(g)).

### 3.9. Immunostaining Images of Genes in Prognostic Models in Normal Renal Tissue and Renal Clear Cell Carcinoma

**Figure 9:**
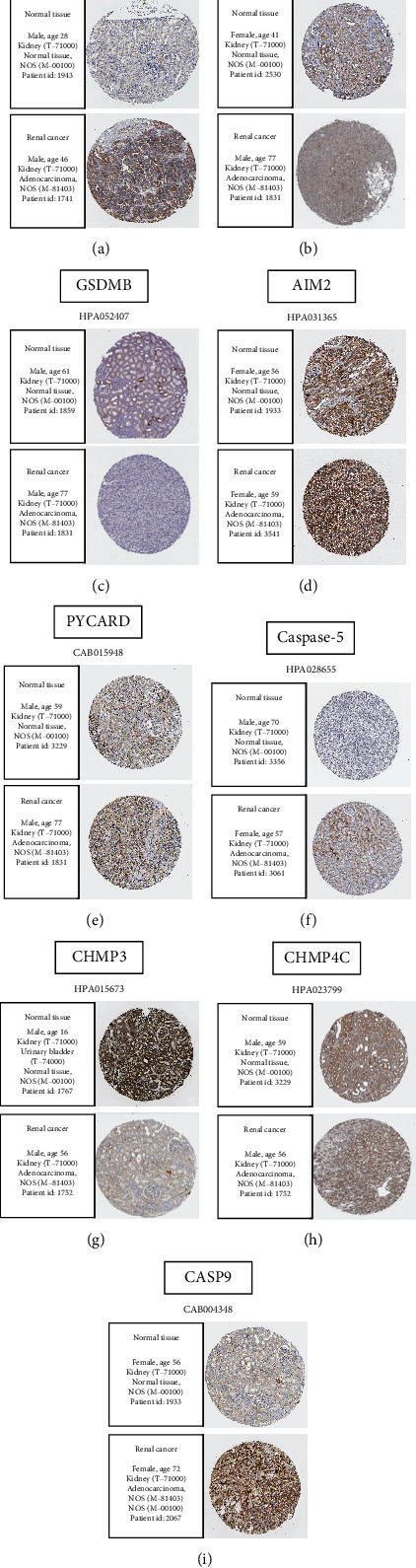
Images of discriminative expression in ccRCC patients as well as normal tissues. Differential coloring pictures of normal renal tissue as well as tumor tissue using antibodies. (a) CASP3. (b) CASP4. (c) GSDMB. (d) AIM2. (e) PYCARD. (f) Caspase-5. (g) CHMP3. (h) CHMP4C. (i) CASP9.

In order to confirm the diagnosis of prognostic genes differentially expressed in renal clear cell carcinoma as well as normal tissues, we utilized HPA database for further validation, as well as consequences are indicated in Figure 9. Compared with normal renal tissues, CASP3, CASP4, Caspase-5, AIM2, CASP9, GSDMB, and PYCARD in renal clear cell carcinoma tissues stained deeper and more widely, showing positive expression (Figures 9(a)–9(b) and 9(i)). Meanwhile, CHMP3 and CHMP4C were significantly stained in normal renal tissues, and the staining range was small in cancer tissues (Figures 9(g) and 9(h)).

### 3.10. Expression of Pyroptosis-Related Genes Correlates with Immune Infiltration

**Figure 10:**
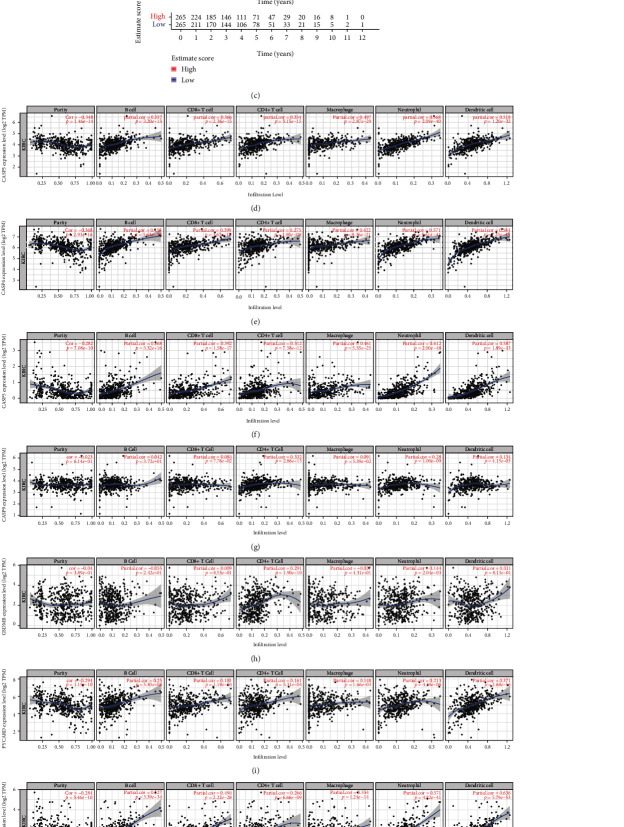
Relationship among prognosis genes as well as immunity infiltrating (TIMER) in microenvironment. (a–c) Contents of each component in the microenvironment and survival curves of ccRCC patients. (d) CASP3. (e) CASP4. (f) CASP5. (g) CASP9. (h) GSDMB. (i) PYCARD. (j) AIM2. (k) CHMP3 (VPS24). (l) CHMP4C.

Inflammatory responses triggered by pyroptosis exert a crucial part in cancer immunity microcircumstance. First, we analyzed the association of stromal components, immune components, and total components in the microenvironment with ccRCC. The consequences indicated only proportion of ImmuneScore could be negatively related to overall survival, and the ratio of other components was not significantly correlated with overall survival (Figures 10(a)–10(c)). Moreover, to further comprehend the relation among nine prognostic genes as well as immunity infiltrating in the ccRCC microcircumstance, the correlation between the expression of prognostic genes (CASP3, CASP4, CASP5, CHMP3 (VPS24), CHMP4C, CASP9, AIM2, GSDMB, and PYCARD) and immunity infiltrating in ccRCC was elucidated with TIMER database. Results showed that CASP3, CASP4, CASP5, and AIM2 expressions in prognostic genes indicated a significant positive relation with macrophage, neutrophil, and dendritic cell in the six immune cells screened, *P* < 0.01 (Figures 10(d)–10(f) and 10(j)). Moreover, expression of AIM2 could be also positively related to B cell and CD8 + T cell, *P* < 0.01 (Figure 10(j)). However, there was little significant correlation between CASP9, GSDMB, CHMP3 (VPS24), CHMP4C, and PYCARD expression and the six immune cells screened (Figures 10(g)–10(i), 10(k), and 10(l)).

## 4. Discussion

In this current paper, we sought to find out prognostic value of pyroptosis-related genes among ccRCC as well as the relationship with tumor immune infiltration and immune checkpoints. We found that the mRNA levels of most of the currently 52 known pyroptosis-related gene relevant genes were discriminatively expressed in ccRCC as well as normal tissues, many of which had single nucleotide variations. Cluster study indicated that C2 could be strongly related to higher renal clear cell carcinoma stage. Univariate and multivariate Cox regression studies indicated risk score was an independent prognosis risk element, as well as our screened prognosis model was able to effectively forecast one-year, three-year, and five-year entire surviving in ccRCC sick persons. A growing number of researches have indicated cellula death mediated by pyroptosis plays a key part in progression of many cancers and can affect all stages of tumors [21]. This mode of cell death is illustrated to be referred to tumor regulation in hepatocellular carcinoma, gastric cancer, breast cancer, and esophageal cancer [22]. A recent report showed that CASP4, CASP5, AIM2, and GSDMB were significantly upregulated in ccRCC, and these upregulated genes could be remarkably related to immune infiltration and poor survival among ccRCC [23–25]. The study also found that models such as CASP3, CASP9, and PYCARD may exert a key part in development of ccRCC, highlighting for the first time the possible role of these genes in ccRCC. Our findings will improve the understanding of PRGs in ccRCC and provide new options for managing and modulating the treatment of ccRCC shortly.

In recent years, pyro-degeneration is a newly recognized type of programmed cell death (PCD). It can be featured via inflammatory corpuscle activating as well as the release of large amounts of inflammatory cytokines, mainly involved in proinflammatory responses [26]. On the one hand, inflammatory responses triggered by pyroptosis exert antitumor effects. On the other hand, excessive inflammation caused by pyroptosis may disrupt immune defense thereby promoting tumor progression [27–29]. Our finding that inflammatory responses triggered by pyroptosis-related genes in the microenvironment lead to poor prognosis in ccRCC patients provides an important reference for understanding prognosis value as well as the underlying link of pyroptosis associated genes among ccRCC. Previous studies have shown that CASP3, CASP9, and PYCARD are involved in tumor invasion and metastasis. For example, CASP3 is reported to be referred to the spread of tumor cellulas in gastrointestinal tumors, and targeted CASP3 therapy can inhibit the development of cancer cells [30]. CASP9 is a protein-coding gene, and many reports have shown that CASP9 gene polymorphisms are associated with increased tumor risk, but this conclusion is controversial [31, 32]. In gliomas, high expression of PYCARD is related to poor patient prognosis and is used as an independent predictor of chemoresistance [33]. However, the model shows high risk is positively associated with low overall survival as well as higher stage, and targeting these genes is a new option for therapy of ccRCC sick persons.

Cancer microcircumstance is a complicated group composition where cancer cellulas recruit large numbers of neutrophils and macrophages to help evade immune surveillance [34, 35]. We found that the prognosis of ccRCC patients was mainly negatively correlated with the immune component, and patients with higher immune proportions had worse prognosis. Function enrichment indicated pyroptosis-related genes could be primarily referred to innate immune regulation such as leukocyte recruitment, neutrophil activation, and activation of inflammatory signaling pathways such as PPAR signaling pathway and Staphylococcus aureus infection. In addition, immune correlation also confirmed that the genes could be primarily referred to macrophage, neutrophil, and dendritic cell regulation. Numerous researches have indicated immunity cellulas in ccRCC microcircumstance have a close link with tumor cells. For example, Braun et al. found a higher proportion of exhausted CD8 + T cells and immunosuppressive M2-like macrophages in patients with advanced ccRCC, and this inhibitory effect showed progressive immune dysfunction as the disease stage of ccRCC progressed [36]. Tessier-Cloutier et al. found that ccRCC microenvironment is infiltrated with a large number of neutrophils, many of which act directly on tumor tissue by releasing elastase thereby promoting tumor spread [37]. The consequences indicate therapy against microenvironmental immunity cellulas can be an efficient strategy for delaying disease progression and activating immune responses.

In addition, our study found significantly higher expression of PDCD1 (PD-1), CTLA4, PDCD1LG2 (PD-L2), TIGIT, and LAG3 (CD223) in populations from the high risking part, which are potential targets for immunity checking point blockade treatment in ccRCC patients. Many researches have indicated T cell function in human cancer microcircumstance is closely related to the ability of T cells to recognize antigens [38]. Influenced by a variety of signaling pathways and cytokines in the TME, only fewer tumor-reactive T cells are functional in the microenvironment. An important driving force is the overexpression of immune checkpoint molecules causing long-lasting antitumor responses that are difficult for T cells to produce [39]. Tumor cells would use the inhibitory checkpoint PD-L1/PD-1 signaling of T cell activity to evade T cell immune killing [40]. Such as in nonsmall cell lung cancer (NSCLC) as well as hepatocellular carcinoma (HCC), a malfunction CD8 + T cell population was discovered to be positively related to high levels of expression of suppressor receptor genes (PD1, PD-L1, LAG3, CTLA4, TIGIT, and HAVCR2) [41, 42]. Zhang et al. found that TIGIT receptor binding to CD155 leads to CD8 + T cell inactivation which supports gastric cancer (GC) development and progression [43]. Therefore, we suspect that in ccRCC patients, pyroptosis produces cytokines that promote immune checkpoint expression while massively recruiting immune cells thereby creating an immunosuppressive microenvironment. However, our study only preliminarily uncovers the relevant link among PGRs as well as tumor immunity microcircumstances, and more prospective studies are required in the future.

Of course, the study has some boundedness. Every analysis was performed with TCGA ccRCC queue, as well as in vitro tests are needed to next identify the consequences. Moreover, the molecular mechanism of PGRs in ccRCC development should be explored.

## 5. Conclusion

In conclusion, we evaluated the diagnostic and prognosis value of cell PGRs in ccRCC sick persons by comprehensive and systematic bioinformatics analysis. Our results may offer novel ideas in the part of pyroptosis-related gene in microenvironment among ccRCC patients, paving way for future assessment of ccRCC prognosis and development of more effective immunotherapeutic strategies.

## Data Availability

RNA sequencing information and corresponding clinic data could be downloaded from TCGA database (https://portal.gdc.cancer.gov/).
